# 2,7-Dibromo-9-octyl-9*H*-carbazole

**DOI:** 10.1107/S1600536808032121

**Published:** 2008-10-22

**Authors:** Eric Gagnon, Dominic Laliberté

**Affiliations:** a Département de Chimie, Université of Montréal, CP 6128, succ. Centre-ville, Montréal, Québec, Canada H3C 3J7; b SolarisChem Inc., 598 Chaline Street, St-Lazare, Québec, Canada J7T 3E8

## Abstract

In the crystal structure of the title compound, C_20_H_23_Br_2_N, the octyl chains are extended in an *anti* conformation and form a segregating bilayer, isolating rows of carbazole units. The carbazole moieties are engaged in offset π–π inter­actions; the smallest centroid-to-centroid distance is 4.2822 (11) Å. This offset packing motif allows the methyl­ene group attached directly to the N atom to be involved in two short C—H⋯π inter­actions (H⋯centroid distances = 2.96 and 2.99 Å) with an adjacent carbazole. One of the Br atoms also participates in a short contact [3.5475 (3) Å] with a symmetry-related (−*x*, 1 − *y*, −*z*) Br atom. This value is significantly smaller than the sum of the van der Waals radii for bromine (3.70 Å).

## Related literature

For general background, see: Morin & Leclerc (2001 ▶). For the structure of 3,6-dibromo-9-hexyl-9*H*-carbazole, see: Duan *et al.* (2005 ▶). For the general use of 2,7-dihalogeno-9-alkyl-9*H*-carbazoles in synthesis, see: Blouin & Leclerc (2008 ▶). For details of halogen⋯halogen inter­actions, see: Desiraju & Parthasarathy (1989 ▶). The synthesis of the title compound was performed according to published procedures (Bouchard *et al.*, 2004 ▶; Dierschke *et al.*, 2003 ▶).
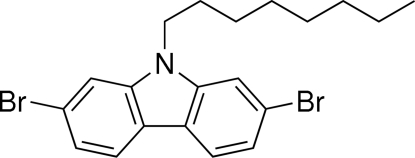


## Experimental

### 

#### Crystal data

C_20_H_23_Br_2_N
*M* *_r_* = 437.21
Monoclinic, 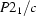

*a* = 20.7256 (4) Å
*b* = 4.6578 (1) Å
*c* = 19.7236 (4) Å
β = 95.945 (1)°
*V* = 1893.79 (7) Å^3^
*Z* = 4
Cu *K*α radiation
μ = 5.40 mm^−1^
*T* = 150 K
0.13 × 0.07 × 0.04 mm

#### Data collection

Bruker Microstar diffractometer
Absorption correction: multi-scan (*SADABS*; Sheldrick, 2007 ▶) *T*_min_ = 0.633, *T*_max_ = 0.806
30701 measured reflections
3301 independent reflections
3158 reflections with *I* > 2σ(*I*)
*R*_int_ = 0.065

#### Refinement

*R*[*F* ^2^ > 2σ(*F* ^2^)] = 0.030
*wR*(*F* ^2^) = 0.083
*S* = 1.07
3301 reflections
209 parameters
H-atom parameters constrained
Δρ_max_ = 0.51 e Å^−3^
Δρ_min_ = −0.39 e Å^−3^

### 

Data collection: *APEX2* (Bruker, 2006 ▶); cell refinement: *SAINT* (Bruker, 2006 ▶); data reduction: *SAINT*; program(s) used to solve structure: *SHELXS97* (Sheldrick, 2008 ▶); program(s) used to refine structure: *SHELXL97* (Sheldrick, 2008 ▶); molecular graphics: *SHELXTL* (Sheldrick, 2008 ▶) and *Material Studio* (Accelrys, 2005 ▶); software used to prepare material for publication: *UdMX* (Maris, 2004 ▶).

## Supplementary Material

Crystal structure: contains datablocks global, I. DOI: 10.1107/S1600536808032121/is2343sup1.cif

Structure factors: contains datablocks I. DOI: 10.1107/S1600536808032121/is2343Isup2.hkl

Additional supplementary materials:  crystallographic information; 3D view; checkCIF report

## Figures and Tables

**Table 1:** Hydrogen-bond geometry (Å, °) *Cg*1 and *Cg*2 are the centroids of the N1/C9–C12 and C5–C10 rings, respectively.

*D*—H⋯*A*	*D*—H	H⋯*A*	*D*⋯*A*	*D*—H⋯*A*
C13—H13*A*⋯*Cg*1^i^	0.98	2.96	3.582 (2)	121
C13—H13*A*⋯*Cg*2^i^	0.98	2.99	3.566 (2)	119

Symmetry code: (i) 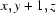.

**Fig. 1.**
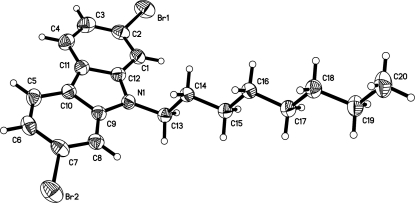
The molecular structure of the title compound. Displacement ellipsoids are drawn at the 50% probability level.

**Fig. 2.**
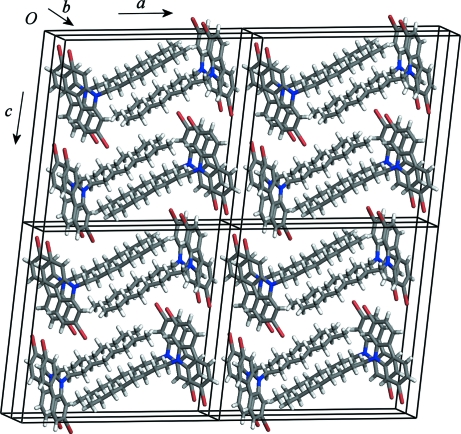
A view of a 2 × 2 × 2 array of unit cells showing 2,7-dibromo-9-octyl-9H-carbazole molecules separated by a bilayer of linear octyl chains.

**Fig. 3.**
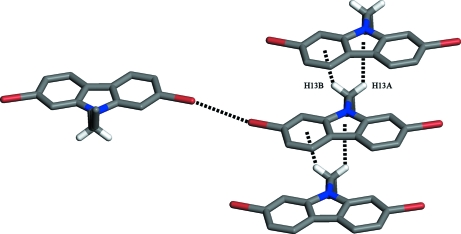
Br···Br contact and C—H···π interactions involving the title compound. All hydrogen atoms except H13A and H13B were removed for clarity.

## supplementary crystallographic information

### Comment

The field of conjugated polymer chemistry is highly dependent on the efficient preparation of suitable monomers. Amongst them, substituted fluorenes, thiophenes and phenylenes are readily accessible, allowing the synthesis of polymers with tailored properties. Until 2001, highly conjugated poly(2,7-carbazoles) could not be prepared because potential precursors such as 2,7-dibromo-9-octyl-9*H*-carbazole were unavailable (Morin & Leclerc, 2001).

In such compounds, an alkyl group is useful because it increases the solubility and helps control molecular packing, which are important parameters in preparing devices such as organic light-emitting diodes and solar cells (Blouin & Leclerc, 2008).

Crystals of 2,7-dibromo-9-octyl-9*H*-carbazole belonging to the space group *P*2_1_/*c* were grown by slowly cooling a saturated hot solution in hexanes. The octyl chain adopts a fully extended conformation, with torsion angles ranging from 174.47 (17)° to 179.9 (2)° (Fig. 1). The octyl groups are parallel and packed tightly, leading to the formation of a bilayered structure (Fig. 2).

The carbazole units pack together through the formation of offset intermolecular π-=π interactions. The smallest centroid···centroid distance is 4.2822 (11) Å and β = 39.81°, which is defined as the angle between the vector *Cg*1 →*Cg*2 and the normal to the least-squares plane of *Cg*1. *Cg*1 and its plane are defined by N1/C9–C12 and *Cg*2 is the centroid of C5–C10. Additional stabilization is provided by C—H···π interactions involving H13A [2.96 Å] and H13B [2.99 Å] (Fig. 3 and Table 1) and short contacts [3.5475 (3) Å] between symmetry-related (-*x*, 1 - *y*, -*z*) bromine atoms (Desiraju & Parthasarathy, 1989). The structure of the related compound, 3,6-dibromo-9-hexyl-9*H*-carbazole, was reported by Duan *et al.* (2005).

### Experimental

The title compound was obtained by a two-step synthesis starting from 4,4'-dibromo-2-nitrobiphenyl. A reductive Cadogan ring-closure reaction was performed according to Dierschke *et al.* (2003) to afford 2,7-dibromocarbazole, which was alkylated with 1-bromooctane following a procedure reported by Bouchard *et al.* (2004). Crystallization of the title compound from hexanes afforded needles which were used in this study. Spectroscopic data proved to be consistent with the reported values.

### Refinement

H atoms were placed in idealized positions and allowed to ride on their parent atoms, with C—H distances of 0.99 Å (methylene), 0.98 Å (methyl) and 0.95 Å (aromatic C—H), and with *U*_iso_(H) of 1.2*U*_eq_(C) for aromatic and methylene H atoms and 1.5*U*_eq_(C) for terminal methyl groups.

### Crystal data

**Table:**

C_20_H_23_Br_2_N	*F*(000) = 880
*M**_r_* = 437.21	*D*_x_ = 1.533 Mg m^−^^3^
Monoclinic, *P*2_1_/*c*	Cu *K*α radiation, λ = 1.54178 Å
Hall symbol: -P 2ybc	Cell parameters from 20371 reflections
*a* = 20.7256 (4) Å	θ = 2.9–67.8°
*b* = 4.6578 (1) Å	µ = 5.40 mm^−^^1^
*c* = 19.7236 (4) Å	*T* = 150 K
β = 95.945 (1)°	Needle, colourless
*V* = 1893.79 (7) Å^3^	0.13 × 0.07 × 0.04 mm
*Z* = 4	

### Data collection

**Table:**

Bruker Microstar diffractometer	3301 independent reflections
Radiation source: Rotating anode	3158 reflections with *I* > 2σ(*I*)
Helios optics	*R*_int_ = 0.065
Detector resolution: 8.3 pixels mm^-1^	θ_max_ = 68.2°, θ_min_ = 4.3°
ω scans	*h* = −24→24
Absorption correction: multi-scan (SADABS; Sheldrick, 2007)	*k* = −5→5
*T*_min_ = 0.633, *T*_max_ = 0.806	*l* = −22→23
30701 measured reflections	

### Refinement

**Table:**

Refinement on *F*^2^	Primary atom site location: structure-invariant direct methods
Least-squares matrix: full	Secondary atom site location: difference Fourier map
*R*[*F*^2^ > 2σ(*F*^2^)] = 0.030	Hydrogen site location: inferred from neighbouring sites
*wR*(*F*^2^) = 0.083	H-atom parameters constrained
*S* = 1.07	*w* = 1/[σ^2^(*F*_o_^2^) + (0.0431*P*)^2^ + 0.9966*P*] where *P* = (*F*_o_^2^ + 2*F*_c_^2^)/3
3301 reflections	(Δ/σ)_max_ = 0.001
209 parameters	Δρ_max_ = 0.51 e Å^−^^3^
0 restraints	Δρ_min_ = −0.38 e Å^−^^3^

### Special details

**Table:**

Experimental. X-ray crystallographic data for the title compound were collected from a single-crystal sample, which was mounted on a loop fiber. Data were collected using a Bruker Microstar diffractometer equipped with a Platinum 135 CCD Detector, Helios optics and a Kappa goniometer. The crystal-to-detector distance was 4.0 cm, and the data collection was carried out in 512 *x* 512 pixel mode. The initial unit-cell parameters were determined by a least-squares fit of the angular setting of strong reflections, collected by a 10.0 degree scan in 33 frames over three different parts of the reciprocal space (99 frames total).Due to geometrical constraints of the instrument and the use of copper radiation, we consistently obtain a data completeness lower than 100% depending on the crystal system and the orientation of the mounted crystal, even with appropriate data collection routines. Typical values for data completeness range from 83–92% for triclinic, 85–97% for monoclinic and 85–98% for all other crystal systems.
Geometry. All e.s.d.'s (except the e.s.d. in the dihedral angle between two l.s. planes) are estimated using the full covariance matrix. The cell e.s.d.'s are taken into account individually in the estimation of e.s.d.'s in distances, angles and torsion angles; correlations between e.s.d.'s in cell parameters are only used when they are defined by crystal symmetry. An approximate (isotropic) treatment of cell e.s.d.'s is used for estimating e.s.d.'s involving l.s. planes.
Refinement. Refinement of *F*^2^ against ALL reflections. The weighted *R*-factor *wR* and goodness of fit *S* are based on *F*^2^, conventional *R*-factors *R* are based on *F*, with *F* set to zero for negative *F*^2^. The threshold expression of *F*^2^ > σ(*F*^2^) is used only for calculating *R*-factors(gt) *etc*. and is not relevant to the choice of reflections for refinement. *R*-factors based on *F*^2^ are statistically about twice as large as those based on *F*, and *R*- factors based on ALL data will be even larger.

### Fractional atomic coordinates and isotropic or equivalent isotropic displacement parameters (Å^2^)

**Table:**

	*x*	*y*	*z*	*U*_iso_*/*U*_eq_	
Br1	0.277870 (14)	0.91023 (7)	0.562136 (14)	0.05940 (12)	
Br2	0.056608 (12)	0.45377 (7)	0.073002 (13)	0.05584 (12)	
C1	0.23693 (9)	0.8734 (4)	0.42106 (11)	0.0366 (5)	
H1	0.2690	1.0155	0.4159	0.044*	
C2	0.22615 (11)	0.7662 (5)	0.48414 (11)	0.0411 (5)	
C3	0.17985 (12)	0.5585 (5)	0.49381 (12)	0.0452 (5)	
H3	0.1744	0.4921	0.5384	0.054*	
C4	0.14194 (11)	0.4495 (4)	0.43831 (12)	0.0405 (5)	
H4	0.1100	0.3080	0.4443	0.049*	
C5	0.07350 (9)	0.2906 (4)	0.27976 (11)	0.0368 (5)	
H5	0.0533	0.1671	0.3096	0.044*	
C6	0.05563 (9)	0.2808 (4)	0.21101 (12)	0.0396 (5)	
H6	0.0231	0.1502	0.1930	0.047*	
C7	0.08542 (10)	0.4637 (4)	0.16739 (12)	0.0377 (5)	
C8	0.13427 (9)	0.6544 (4)	0.19041 (10)	0.0340 (4)	
H8	0.1547	0.7739	0.1600	0.041*	
C9	0.15163 (8)	0.6612 (4)	0.26027 (10)	0.0296 (4)	
C10	0.12158 (9)	0.4832 (4)	0.30598 (11)	0.0313 (4)	
C11	0.15084 (9)	0.5491 (4)	0.37300 (11)	0.0333 (4)	
C12	0.19845 (9)	0.7622 (4)	0.36566 (10)	0.0317 (4)	
C13	0.24263 (9)	1.0219 (4)	0.26789 (10)	0.0312 (4)	
H13A	0.2547	1.1795	0.3004	0.037*	
H13B	0.2208	1.1080	0.2257	0.037*	
C14	0.30385 (9)	0.8674 (4)	0.25132 (11)	0.0319 (4)	
H14A	0.3277	0.7976	0.2942	0.038*	
H14B	0.2914	0.6981	0.2226	0.038*	
C15	0.34861 (9)	1.0575 (4)	0.21437 (11)	0.0327 (4)	
H15A	0.3241	1.1362	0.1728	0.039*	
H15B	0.3631	1.2212	0.2442	0.039*	
C16	0.40776 (9)	0.8980 (4)	0.19451 (11)	0.0341 (4)	
H16A	0.3931	0.7254	0.1680	0.041*	
H16B	0.4339	0.8325	0.2365	0.041*	
C17	0.45074 (10)	1.0767 (4)	0.15261 (12)	0.0364 (5)	
H17A	0.4241	1.1515	0.1119	0.044*	
H17B	0.4676	1.2434	0.1801	0.044*	
C18	0.50750 (10)	0.9090 (4)	0.12985 (12)	0.0396 (5)	
H18A	0.5331	0.8288	0.1706	0.048*	
H18B	0.4904	0.7457	0.1013	0.048*	
C19	0.55238 (10)	1.0841 (5)	0.08983 (13)	0.0442 (5)	
H19A	0.5698	1.2473	0.1182	0.053*	
H19B	0.5270	1.1638	0.0488	0.053*	
C20	0.60875 (12)	0.9104 (6)	0.06785 (16)	0.0586 (7)	
H20A	0.6350	0.8366	0.1083	0.088*	
H20B	0.6356	1.0334	0.0418	0.088*	
H20C	0.5920	0.7494	0.0393	0.088*	
N1	0.19774 (7)	0.8298 (3)	0.29719 (8)	0.0309 (3)	

### Atomic displacement parameters (Å^2^)

**Table:**

	*U*^11^	*U*^22^	*U*^33^	*U*^12^	*U*^13^	*U*^23^
Br1	0.06460 (19)	0.0781 (2)	0.03306 (19)	0.00348 (13)	−0.00678 (13)	−0.00578 (11)
Br2	0.04915 (17)	0.0795 (2)	0.03734 (19)	0.00033 (11)	−0.00278 (13)	−0.01405 (11)
C1	0.0320 (9)	0.0419 (10)	0.0357 (12)	0.0036 (8)	0.0031 (9)	−0.0021 (8)
C2	0.0427 (11)	0.0501 (12)	0.0298 (12)	0.0099 (9)	0.0008 (9)	−0.0031 (9)
C3	0.0529 (13)	0.0517 (12)	0.0328 (13)	0.0090 (10)	0.0138 (11)	0.0056 (9)
C4	0.0428 (11)	0.0422 (11)	0.0385 (13)	0.0019 (8)	0.0140 (10)	0.0047 (9)
C5	0.0302 (9)	0.0356 (9)	0.0458 (13)	−0.0001 (8)	0.0101 (9)	−0.0009 (9)
C6	0.0286 (9)	0.0395 (10)	0.0507 (14)	−0.0002 (8)	0.0045 (9)	−0.0085 (9)
C7	0.0311 (10)	0.0460 (11)	0.0356 (12)	0.0081 (8)	0.0019 (9)	−0.0082 (9)
C8	0.0297 (9)	0.0389 (10)	0.0338 (12)	0.0039 (8)	0.0049 (8)	0.0005 (8)
C9	0.0255 (8)	0.0324 (9)	0.0315 (11)	0.0041 (7)	0.0052 (8)	−0.0006 (7)
C10	0.0269 (9)	0.0334 (9)	0.0346 (12)	0.0049 (7)	0.0077 (8)	0.0008 (8)
C11	0.0301 (9)	0.0351 (9)	0.0359 (12)	0.0045 (7)	0.0097 (9)	0.0015 (8)
C12	0.0295 (9)	0.0358 (9)	0.0305 (11)	0.0055 (7)	0.0068 (8)	0.0014 (8)
C13	0.0302 (9)	0.0325 (9)	0.0314 (11)	0.0002 (7)	0.0051 (8)	0.0018 (8)
C14	0.0293 (9)	0.0334 (9)	0.0333 (11)	0.0014 (7)	0.0045 (8)	0.0037 (8)
C15	0.0301 (9)	0.0332 (9)	0.0352 (12)	0.0004 (7)	0.0051 (9)	0.0032 (8)
C16	0.0297 (9)	0.0367 (9)	0.0362 (12)	0.0016 (7)	0.0049 (9)	0.0038 (8)
C17	0.0316 (9)	0.0381 (10)	0.0401 (13)	0.0006 (8)	0.0067 (9)	0.0032 (8)
C18	0.0344 (10)	0.0407 (10)	0.0450 (14)	0.0008 (8)	0.0103 (10)	0.0030 (9)
C19	0.0365 (11)	0.0469 (12)	0.0509 (15)	−0.0043 (9)	0.0133 (10)	0.0010 (10)
C20	0.0441 (13)	0.0647 (15)	0.071 (2)	−0.0028 (11)	0.0275 (13)	−0.0028 (13)
N1	0.0282 (7)	0.0360 (8)	0.0289 (9)	−0.0009 (6)	0.0050 (7)	0.0017 (7)

### Geometric parameters (Å, °)

**Table:**

Br1—C2	1.904 (2)	C13—C14	1.523 (2)
Br2—C7	1.896 (2)	C13—H13A	0.9900
C1—C2	1.380 (3)	C13—H13B	0.9900
C1—C12	1.385 (3)	C14—C15	1.522 (2)
C1—H1	0.9500	C14—H14A	0.9900
C2—C3	1.390 (3)	C14—H14B	0.9900
C3—C4	1.376 (4)	C15—C16	1.519 (3)
C3—H3	0.9500	C15—H15A	0.9900
C4—C11	1.399 (3)	C15—H15B	0.9900
C4—H4	0.9500	C16—C17	1.524 (3)
C5—C6	1.369 (3)	C16—H16A	0.9900
C5—C10	1.399 (3)	C16—H16B	0.9900
C5—H5	0.9500	C17—C18	1.518 (3)
C6—C7	1.399 (3)	C17—H17A	0.9900
C6—H6	0.9500	C17—H17B	0.9900
C7—C8	1.387 (3)	C18—C19	1.518 (3)
C8—C9	1.388 (3)	C18—H18A	0.9900
C8—H8	0.9500	C18—H18B	0.9900
C9—N1	1.384 (3)	C19—C20	1.520 (3)
C9—C10	1.416 (3)	C19—H19A	0.9900
C10—C11	1.429 (3)	C19—H19B	0.9900
C11—C12	1.417 (3)	C20—H20A	0.9800
C12—N1	1.385 (2)	C20—H20B	0.9800
C13—N1	1.453 (2)	C20—H20C	0.9800
			
C2—C1—C12	116.24 (19)	C13—C14—H14A	109.0
C2—C1—H1	121.9	C15—C14—H14B	109.0
C12—C1—H1	121.9	C13—C14—H14B	109.0
C1—C2—C3	123.7 (2)	H14A—C14—H14B	107.8
C1—C2—Br1	118.09 (17)	C16—C15—C14	112.77 (15)
C3—C2—Br1	118.25 (17)	C16—C15—H15A	109.0
C4—C3—C2	119.5 (2)	C14—C15—H15A	109.0
C4—C3—H3	120.2	C16—C15—H15B	109.0
C2—C3—H3	120.2	C14—C15—H15B	109.0
C3—C4—C11	119.4 (2)	H15A—C15—H15B	107.8
C3—C4—H4	120.3	C15—C16—C17	113.92 (16)
C11—C4—H4	120.3	C15—C16—H16A	108.8
C6—C5—C10	119.78 (18)	C17—C16—H16A	108.8
C6—C5—H5	120.1	C15—C16—H16B	108.8
C10—C5—H5	120.1	C17—C16—H16B	108.8
C5—C6—C7	119.84 (19)	H16A—C16—H16B	107.7
C5—C6—H6	120.1	C18—C17—C16	113.20 (16)
C7—C6—H6	120.1	C18—C17—H17A	108.9
C8—C7—C6	122.8 (2)	C16—C17—H17A	108.9
C8—C7—Br2	118.81 (16)	C18—C17—H17B	108.9
C6—C7—Br2	118.36 (16)	C16—C17—H17B	108.9
C7—C8—C9	116.41 (18)	H17A—C17—H17B	107.8
C7—C8—H8	121.8	C19—C18—C17	114.38 (17)
C9—C8—H8	121.8	C19—C18—H18A	108.7
N1—C9—C8	128.98 (17)	C17—C18—H18A	108.7
N1—C9—C10	108.81 (17)	C19—C18—H18B	108.7
C8—C9—C10	122.21 (18)	C17—C18—H18B	108.7
C5—C10—C9	118.90 (19)	H18A—C18—H18B	107.6
C5—C10—C11	134.20 (18)	C18—C19—C20	113.13 (19)
C9—C10—C11	106.90 (17)	C18—C19—H19A	109.0
C4—C11—C12	119.1 (2)	C20—C19—H19A	109.0
C4—C11—C10	134.14 (19)	C18—C19—H19B	109.0
C12—C11—C10	106.81 (17)	C20—C19—H19B	109.0
C1—C12—N1	129.14 (18)	H19A—C19—H19B	107.8
C1—C12—C11	122.11 (18)	C19—C20—H20A	109.5
N1—C12—C11	108.76 (18)	C19—C20—H20B	109.5
N1—C13—C14	112.13 (15)	H20A—C20—H20B	109.5
N1—C13—H13A	109.2	C19—C20—H20C	109.5
C14—C13—H13A	109.2	H20A—C20—H20C	109.5
N1—C13—H13B	109.2	H20B—C20—H20C	109.5
C14—C13—H13B	109.2	C9—N1—C12	108.71 (15)
H13A—C13—H13B	107.9	C9—N1—C13	125.15 (16)
C15—C14—C13	113.00 (15)	C12—N1—C13	125.82 (17)
C15—C14—H14A	109.0		
			
C12—C1—C2—C3	0.0 (3)	C9—C10—C11—C12	−0.84 (19)
C12—C1—C2—Br1	179.77 (14)	C2—C1—C12—N1	179.21 (18)
C1—C2—C3—C4	0.0 (3)	C2—C1—C12—C11	−0.4 (3)
Br1—C2—C3—C4	−179.72 (16)	C4—C11—C12—C1	0.6 (3)
C2—C3—C4—C11	0.3 (3)	C10—C11—C12—C1	−179.15 (17)
C10—C5—C6—C7	−0.1 (3)	C4—C11—C12—N1	−179.03 (16)
C5—C6—C7—C8	1.5 (3)	C10—C11—C12—N1	1.2 (2)
C5—C6—C7—Br2	−177.43 (14)	N1—C13—C14—C15	174.47 (17)
C6—C7—C8—C9	−1.5 (3)	C13—C14—C15—C16	−176.84 (18)
Br2—C7—C8—C9	177.42 (13)	C14—C15—C16—C17	175.50 (18)
C7—C8—C9—N1	−179.35 (17)	C15—C16—C17—C18	−176.75 (19)
C7—C8—C9—C10	0.2 (3)	C16—C17—C18—C19	−178.2 (2)
C6—C5—C10—C9	−1.1 (3)	C17—C18—C19—C20	179.9 (2)
C6—C5—C10—C11	179.62 (19)	C8—C9—N1—C12	−179.81 (18)
N1—C9—C10—C5	−179.27 (16)	C10—C9—N1—C12	0.58 (19)
C8—C9—C10—C5	1.1 (3)	C8—C9—N1—C13	−6.1 (3)
N1—C9—C10—C11	0.18 (19)	C10—C9—N1—C13	174.33 (16)
C8—C9—C10—C11	−179.46 (16)	C1—C12—N1—C9	179.27 (18)
C3—C4—C11—C12	−0.5 (3)	C11—C12—N1—C9	−1.12 (19)
C3—C4—C11—C10	179.1 (2)	C1—C12—N1—C13	5.6 (3)
C5—C10—C11—C4	−1.2 (4)	C11—C12—N1—C13	−174.82 (16)
C9—C10—C11—C4	179.4 (2)	C14—C13—N1—C9	−86.0 (2)
C5—C10—C11—C12	178.5 (2)	C14—C13—N1—C12	86.7 (2)

### Hydrogen-bond geometry (Å, °)

**Table:**

*D*—H···*A*	*D*—H	H···*A*	*D*···*A*	*D*—H···*A*
C13—H13A···Cg1^i^	0.98	2.96	3.582 (2)	121
C13—H13A···Cg2^i^	0.98	2.99	3.566 (2)	119

Symmetry codes: (i) *x*, *y*+1, *z*.
